# Volume-targeted ventilation vs pressure-controlled ventilation for very low birthweight infants: a protocol of a randomized controlled trial

**DOI:** 10.1186/s13063-023-07564-x

**Published:** 2023-08-16

**Authors:** Jun Tang, Lingyue Gong, Tao Xiong, Chao Chen, Ke Tian, Aoyu Wang, Yi Huang, Wenli Liu, Rong Zhou, Jun Zhu, Dezhi Mu

**Affiliations:** 1 Key Laboratory of Birth Defects and Related Diseases of Women and Children, Ministry of Education, West China Second University Hospital, Sichuan University, Renmin South Road #16, Wuhou District, Chengdu, China; 2 West China Second University Hospital, Sichuan University, Renmin South Road #20, Wuhou District, Chengdu, China

**Keywords:** Volume-targeted ventilation, Pressure-controlled ventilation, Very low-birth-weight infant, Bronchopulmonary dysplasia, Mortality, Mechanical ventilation

## Abstract

**Background:**

Mechanical ventilation (MV) is essential in the management of critically ill neonates, especially preterm infants. However, inappropriate or prolonged use of invasive MV may result in ventilator-associated lung injury. A systemic review comparing pressure control ventilation (PCV) with volume-targeted ventilation mode (VTV) approved that VTV reduces the incidence of death or bronchopulmonary dysplasia (BPD) in neonates; however, this study did not analyze subgroups of very low birthweight (VLBW) infants. Therefore, the aim of this study was to compare the use of VTV and PCV in VLBW infants and to provide clinical evidence for reducing mortality and complications of MV in VLBW infants.

**Method:**

A single-center randomized controlled trial will be performed. All eligible infants will be randomized and assigned to either VTV or PCV group with 1:1 ratio using sealed envelopes. Death or BPD at 36 weeks’ postmenstrual age will be used as the primary outcome. Secondary outcomes include BPD, death, length of invasive MV, noninvasive mechanical ventilation, and oxygen use, length of hospital stay, failure of conventional MV, rate of using high-frequency oscillatory ventilation (HFOV) as rescue therapy, rate of reintubation within 48 h, and hospital expenses.

**Discussion:**

Systemic review suggested that VTV decreases the incidence of death or BPD in neonates compared to PLV; however, this study did not specifically analyze subgroups of VLBW infants. We designed this single-center randomized controlled trials (RCT) to add a significant contribution regarding the benefits of VTV for VLBW patients.

**Supplementary Information:**

The online version contains supplementary material available at 10.1186/s13063-023-07564-x.

## Introduction

Mechanical ventilation(MV) is essential in the management of critically ill neonates, especially preterm infants. Seventy-six percent of preterm infants at 26–28 weeks of gestational age require invasive MV; 33% and 16% of preterm infants at 29–32 weeks and 33–34 weeks of gestational age, respectively, require invasive MV. However, inappropriate or prolonged use of invasive MV may result in ventilator-associated lung injury [1, 2] and be associated with the high risk of bronchopulmonary dysplasia (BPD) [3–5]. Therefore, it is important to select a better ventilation mode for neonates.

Pressure control ventilation (PCV) or sometimes called pressure limited ventilation (PLV) is an effective mode for newborn infants who need respiratory support. Each breath is delivered with a preset peak inspiratory pressure (PIP), which prevents barotrauma caused by high pressure. However, tidal volume (Vt) is varied with patient’s inspiratory effort, lung compliance, and airway resistance. Actual delivered Vt appears to be too large or too small, resulting in over- or under-ventilation in the mode of PCV [6].

Volume-guaranteed (VG) ventilation is a type of volume-targeted ventilation mode (VTV), which can automatically adjust the inspiratory pressure within the set maximum pressure, in order to deliver Vt based on the pre-set target Vt [7]. In VTV, actual Vt stays relatively stable to better stabilize the partial pressure of carbon dioxide(PCO_2_) [8, 9]. When the infants’ lung condition improves, the ventilator weans the inspiratory pressure downward in real-time according to the target Vt to reduce the mean airway pressure(MAP), thus reducing the length of MV [10].

An international survey published in 2011 found that the use of VTV in neonates is gradually increasing. Approximately 50% of the included institutions use VTV as their routine mode [11]. Nevertheless, the clinical use of VTV in neonates varies in different countries. A survey of neonatologists in Canada and the USA published in 2019 found that the use of VTV was higher than in Canada (81%) than in the USA (39%). And the main reasons for not using VTV was “lack of understanding” [12].

A 2017 Cochrane review of 20 randomized controlled trials (RCT) comparing PLV with VTV in 1065 neonates approved that VTV reduces the incidence of death or BPD in neonates [10]; however, this study did not analyze subgroups of very low birthweight (VLBW) infants. A meta-analysis found that the use of VTV did not increase in adverse events, suggesting that the use of VTV in extremely low birthweight (ELBW) is safe [13].

In the latest 20 years, evidence about VTV as primary respiratory support in VLBW is few. One study reported that using VTV in infants with birthweights of 500–1249 g did not alter time to extubation [14]. However, this study was done in 2005. Another RCT with small sample size found that VTV decreased combined outcome of BPD or death [15]. More high-quality clinical evidence of VTV is needed. Therefore, the aim of this study was to compare the use of VTV and PCV in VLBW infants and to provide clinical evidence for reducing mortality and complications of MV in VLBW infants.

## Methods: participants, interventions and outcomes

### Objective

The objective is as follows: to investigate the effects of VTV and PLV as a respiratory support mode on BPD/death in VLBW infants.

### Study design

A single-center randomized controlled trial will be performed. Patients will be randomized in two parallel groups: VTV vs PCV in 1:1 ratio. The study type was a superiority study to verify the use of VTV over PCV in VLBW infants.

### Inclusion criteria

Infants satisfying the following inclusion criteria will be eligible to participate:

1. Admitted for admission to neonatal intensive care unit, and
2. Birthweight < 1500 g, and
3. Infant need invasive mechanical ventilation.

### Exclusion criteria

Infants with the following exclusion criteria will not be eligible to participate:

1. Fatal congenital malformations or chromosome abnormality found before or after randomization, or
2. Diaphragmatic hernia, meconium aspiration syndrome, and air leak syndrome, or
3. High-frequency oscillatory ventilation (HFOV) or any other invasive mode has been used before, or
4. Refusal to participate in the trial.

### Sample size

An RCT of VLBW infants with respiratory distress syndrome(RDS) receiving VTV or PCV as their primary respiratory support mode showed that the BPD/death rate of 26% and 64% respectively [15]. Based on this study, a sample size of 31 in each group will have 90% power to detect statistically difference at a 0.05 tow-sided significance level (MedSci, Shanghai, China). Considering a 20% of patients may withdraw from the research, a sample size of 68 will be appropriate.

### Recruitment

This study will be implemented in West China Second Hospital, a teaching hospital in Chengdu, China.

After admission of all infants who might meet the eligibility, a written informed consent will be explained to at least one parent or legal guardian by the principal investigator or delegated deputy. Informed consent will be signed by at least one parent or legal guardian. All patient admitted in our neonatal intensive care unit (NICU) will be screened. Based on patient in-charged in 2022, we anticipate 1 year to recruit enough patients. This time might be prolonged or shortened due to different reasons. A senior investigator will be available at all times to discuss concerns raised by parents or clinicians during the course of the trial.

## Assignment of interventions: allocation

Randomization will be immediately performed once the eligibility is confirmed. All eligible infants will be randomized and assigned to either VTV or PCV group with 1:1 ratio. Stratified randomization will be done according to a computer-generated random number and variable block sizes (2) will be used to strengthen allocation concealment. Multiple birth infants will be randomized separately. Randomization will be stratified by birthweight (< 1000 g versus > / = 1000 g).

### Implementation

The second author will generate the allocation sequence. All authors will participate in enrolling and assigning participants. The names of the treatment groups were placed into envelopes per the randomization sequence, sealed, and labeled with a sequential participant number. Once a patient consented to participate, a researcher who is on call that time will open the envelope to reveal the name of the assigned group.

## Assignment of interventions: blinding

Both of the modes have parameter adjustment on the ventilator; respiratory therapists and attending physicians will not be blinded due to the nature of the study. Blinding was set for the remaining study personnel like statistician, patients, and families. There will be no circumstances under which unblinding is permissible, as the respiratory therapists will not be blinded due to the nature of the study and family members are not allowed to get into the NICU due to the hospital policy. To minimize bias, “ventilator adjusting protocol” and “criteria for extubation” have been carefully defined and adhered.

### Primary outcome

Death or BPD at 36 weeks’ postmenstrual age will be used as the primary outcome. BPD will be diagnosed by clinical physicians. The definition and diagnostic criteria of BPD is according to NIH 2018 [16]. We have chosen “death or BPD (36w)” as our primary outcome to maintain similarity with previous trial comparing VTV and PCV [10].

### Secondary outcome

1. BPD
2. Death
3. Length of invasive MV, noninvasive mechanical ventilation, and oxygen use
4. Length of hospital stay
5. Failure of conventional MV. Rate of using HFOV as rescue therapy
  - Criteria for the failure of conventional MV (meet any one or more of these points):
    i. Infants with 100% inhaled oxygen concentration and MAP ≥ 15 cmH_2_O have oxygen saturation (SpO_2_) less than 90% or two blood gasses monitored at least 2 h apart show partial pressure of oxygen (PaO_2_) less than 50 mmHg
    ii. Inability to maintain PCO_2_ < 60 mmHg and pH < 7.25 with conventional MV mode
    iii. Clinical manifestations confirming unsuitability for use of the current mode, for example, air leak syndrome and severe pulmonary hemorrhage, etc.
    iv. The clinical team consider the infant’s current clinical condition unsuitable for the continuation of the current mode of ventilation
6. Rate of reintubation within 48 h
7. The risk of complications: air leak syndrome, grades 3 to 4 intraventricular hemorrhage (IVH), periventricular leukomalacia (PVL), stage 3 or higher retinopathy of prematurity (ROP), necrotizing enterocolitis (NEC), pulmonary hemorrhage
8. Hospital expenses

### Other collected data

The following data will be recorded for each infant: demographic information: patient’s record number, gestational age, gender, birthweight, multiple birth, delivery way, antenatal steroid treatment, preterm premature rupture of membranes, chorioamnionitis, maternal eclampsia; baseline information: Apgar score at 1, 5, 10 min, amniotic fluid status, times of using supplementary pulmonary surfactant, small for gestational age, age at intubation (h); other information: ventilator settings and measurements including MAP, fraction of inhaled oxygen concentration (FiO_2_), PIP, Vt, respiratory rate (RR), and minute ventilation. Other vital signs and arterial blood gas (ABG) results including SpO_2_, PaO_2_, pH, PCO_2_, oxygen index, and heart rate.

### Participant timeline

See Fig. 1.

**Fig. 1:**
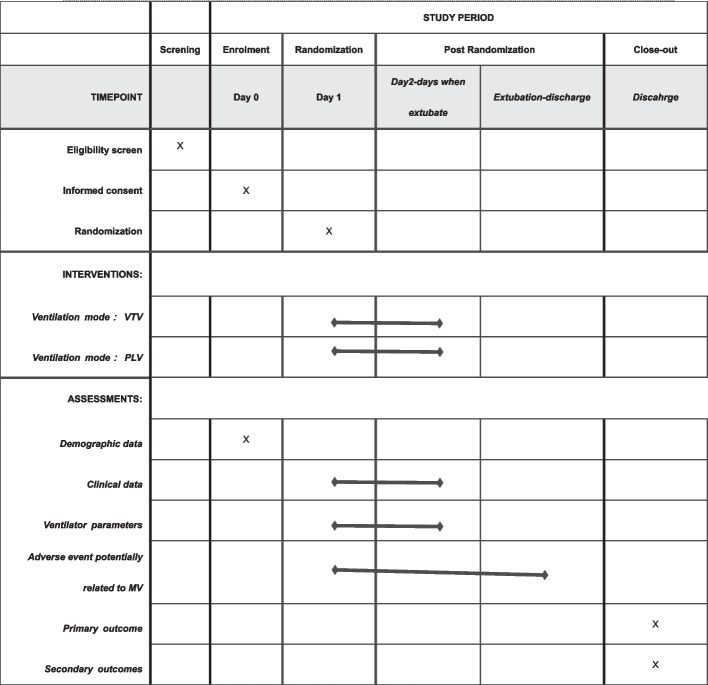
Schedules of assessment and procedures

### Intervention

#### Ventilator

Evita V300, Evita V500 (with volume guaranteed module, Draeger, Lubeck, Germany) or Fabian (ACUTRONIC Medical System, Hirzel, Switzerland) will be used depending on which one is available at that time.

**Fig. 2:**
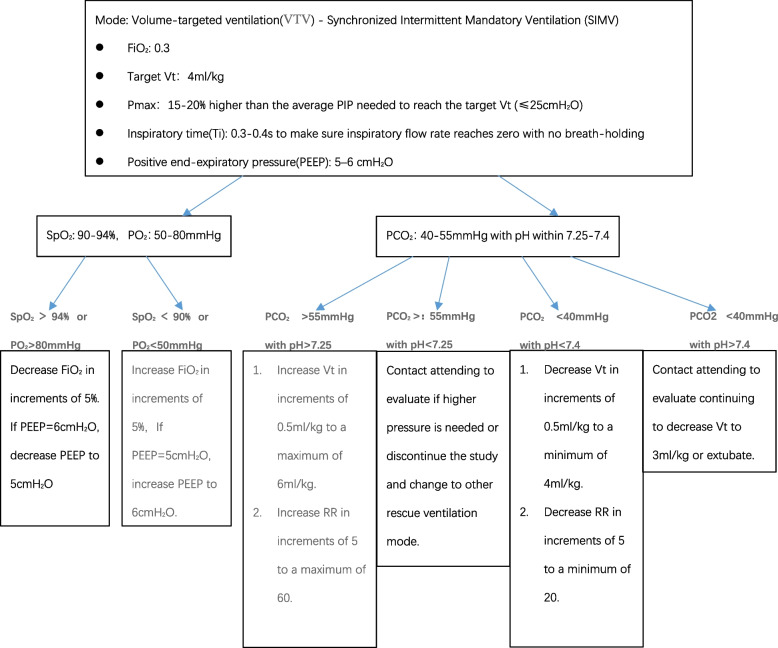
Volume-targeted ventilation protocol for very low birthweight infants

Initial ventilator settings and parameter adjustment (Fig. 2).

**Fig. 3:**
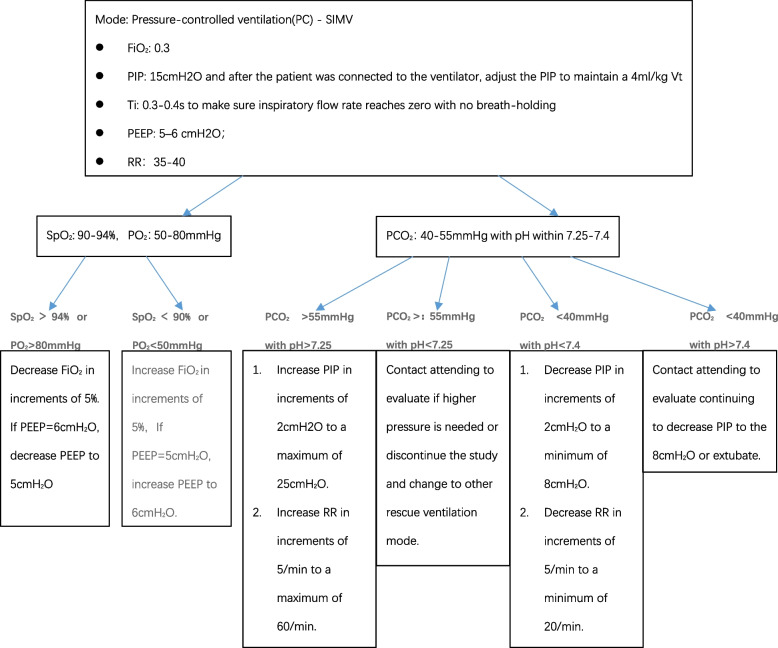
Pressure-controlled ventilation protocol for very low birthweight infants

Retest ABG or transcutaneous PCO_2_ 2 h after adjusting parameters (Fig. 3).

Retest ABG or transcutaneous PCO_2_ 2 h after adjusting parameters.

### Extubation criteria

The extubation criteria are as follows: infant’s MAP ≤ 10 cmH_2_O without tachypnea or labored breathing, current weight ≥ 80% of birthweight, and infant has adequate spontaneous breathing.

### Strategies to improve adherence to interventions

To improve adherence to research interventions, a flow sheet will be displayed at each bedside as a reminder to the respiratory therapists or physicians of the allowed parameter adjustment.

### Routine care after extubation

Caffeine could be used to prevent apnea.

Nasal intermittent positive pressure ventilation will be used after extubation.

Re-intubate if clinically indicated.

If the infant was reintubated, the original randomly assigned ventilation mode should be used.

## Data collection and management

### Data collection

Data will be collected on a web-based electronic case record form by research investigators. Some collected data can be obtained from the clinical records, and the rest will be collected from the ventilator at the bedside. Clinical information will be collected in three forms at the following times: demographic information will be collected at trial entry, baseline information will be collected after randomization, and outcome information: including primary outcomes, secondary outcomes, and others. Outcomes will be collected before discharge. And other observational information like ABG, ventilator settings, and respiratory mechanics will be collected 1 h after using the ventilator and same time each day until extubation.

Any participants who discontinue or deviated from the intervention protocol has to collect the specific reason.

### Confidentiality

The data safety monitoring board (DSMB) will be responsible for the preservation of data as well as the verification of data authenticity and correctness to ensure the accuracy of study results.

All participants will be identified using a reference number that will be used for the entire duration of the study. All data are needed to be strictly secured during transmission as well as archiving to ensure patient privacy and prevent data leakage or loss. All information that contains names or other personal identifiers, such as locator forms and informed consent forms, will be stored separately from study records identified by number, and contact information should be left after obtaining the family’s consent for the subsequent follow-up study, if any. The participant information materials and informed consent form are available from the corresponding author on request.

All data need to be kept for at least 3 years after the completion of the trial.

### Statistical methods

All efficacy analysis will be conducted on an intention-to-treat basis. The SPSS version 26.0 (SPSS, Chicago, IL) software will be used for statistical analysis. Clinical characteristics of infants will be described as mean with standard deviation or median with range for continuous variables and rate or percentage for categorical variables. Absolute risk differences with 95% confidence intervals (CIs) between VTV and PCV will be calculated for binary outcomes, and odds ratios with 95% CIs will be estimated by univariate logistic regression, followed by multivariate logistic regression if the prespecified baseline characteristics are unbalanced between the two groups. Absolute mean differences with 95% CIs (using *t*-test) or mean differences with P25-P75 (using Hodges-Lehmann) will be also calculated for continuous outcomes, and adjusted differences will be estimated by multivariate liner regression if needed. A *P* value < 0.05 was considered statistically significant.

### Interim analyses

There are no plans to conduct an interim analysis of the outcomes.

### Methods for additional analyses (e.g., subgroup analyses)

Analyses will be performed in the birthweight less than 1000 g and birthweight of 1000 g or more subgroups to assess the homogeneity of the difference between the two treatment groups. These subgroup analyses will be supplemented by interaction tests between the treatment and the subgroup variables. The results will be graphically presented using Forest plots.

### Methods in analysis to handle protocol non‐adherence and any statistical methods to handle missing data

Patients with missing outcome data will be censored at last contact. Descriptive statistics on the use of non-protocolized modes as well as intolerance of conventional ventilation mode (duration in days and percentage of time in study spent on HFOV) will be provided.

## Oversight and monitoring

### Composition of the coordinating center and trial steering committee

The research project members will meet weekly throughout the study. The executive steering committee (ESC) will consist of the lead investigators (LYG, JT, CC, KT, AYW) with support from the study statistician (TX). The ESC will supervise all aspects of the study, for example, implementation of all policies and the daily operations.

### Composition of the data monitoring committee, its role and reporting structure

A DSMB will ensure patient data safety during the study and oversee the overall conduct of the trial. The DSMB consisting of two study members who are independent from funder or sponsor, who both have no competing interests to declare, will be responsible for the preservation of data as well as the verification of data authenticity and correctness to ensure the accuracy of study results.

After 10 patients are included, the DSMB will assess the occurrence of adverse events in relation to the study’s relevance and discuss with the study team whether the intervention needs to be modified.

### Harms

Although we do not anticipate any additional risks associated with study participation, the following monitoring methods will be used to ensure patient safety. All serious or unexpected adverse events that are potentially causally related to the intervention, or are considered potentially directly related to study participation (i.e., unlikely to occur if the patient does not participate in the study), will be reported to the CC and monitor via an electronic case report form and reviewed by the DSMB at the meeting. Serious adverse events (SAE) should be documented in the source and SAE. Investigators should promptly report SAEs that are potentially or clearly relevant to the study intervention within 24 h of learning of the event and update additional information as it becomes available. Each site is also required to report SAEs that are potentially or clearly related to the study intervention to the ethic board.

### Auditing

The research project members will meet weekly throughout the study to report the implementation of the study. ESC will meet when accrued 10 patients to review trial conduct. Source data verification will be performed on a selection of the patients by comparing the data in patient’s record with the data collected. For selected participants, the presence of signed informed consent and compliance with inclusion and exclusion criteria will be checked.

### Protocol amendments

If there are any modifications in the protocol during the study, including changes to study design, eligibility criteria, outcomes, analyses, patient population, sample sizes, study procedures, a formal amendment to the protocol is required and submitted to the ethics committee for review.

### Dissemination plans

The results of the study will be published in peer-reviewed journals and presented at national and international conferences. Communication, reporting, and publication of the study results will be the responsibility of the study’s principal investigator and steering committee.

## Discussion

A follow-up study of VLBW infants (*n* = 270) who had been treated with VTV or PLV after birth found that, in the short term, VTV reduced the incidence of interstitial lung disease during hospitalization, length of hospital stay, and mortality rates. Long-term follow-up found fewer deaths or neurological injuries in the VTV group compared with PLV [17]. A meta-analysis found that for ELBW infants (*n* = 247), there were no statistically significant differences between VTV and PLV in the mortality, BPD, length of MV, hypocapnia, FiO_2_, IVH/ PVL, and air leak syndrome. The use of VTV did not increase in adverse events, suggesting that the use of VTV in ELBW is safe [13]. However, evidence is lacking regarding the benefits of VTV in VLBW. It is important to provide an RCT to help better understand this mode in VLBW. We carefully designed this single-center RCT. We chose BPD or death at 36 weeks’ postmenstrual age as our primary outcome. In addition to that, we will also collect ventilator adjustments, lung mechanics, and other secondary outcomes. The study will pay special attention to ventilator-associated lung injury. We hope this trial to add a significant contribution regarding the benefits of VTV for VLBW patients.

In conclusion, we describe a protocol for a single-center RCT in VLBW patients to compare two ventilator modes, VTV and PLV.

## Definitions

Withdrawal: Patients died or were discharged early due to non-respiratory causes (e.g., infectious shock, cardiac insufficiency, new congenital anomalies discovered after inclusion in the study, family wishes, etc.). All withdrawn patients need to record the specific reason for withdrawal.

Lost to follow-up: Difficult to follow-up after discharge due to family wishes or loss of contact information.

Discontinuation: Patients requiring higher respiratory support/ECMO due to difficulty maintaining oxygenation with the ventilator parameters established by protocol; all patients discontinuing the study should be evaluated by the attending, and the specific reasons and ventilator parameters should be documented by the investigator.

Adverse events/side effects: adverse events that may be caused by the intervention, such as air-leak syndrome, ventilator-associated pneumonia (VAP), and unplanned extubation. We will report all adverse events. If patient diagnosed with air-leak syndrome after enrollment, we will evaluate placing a chest tube depend on the hospital protocol. If unplanned extubation happened, we will evaluate patient’s respiratory condition to decide whether reintubate or not. If an adverse event such as unplanned extubation or pneumothorax occurs leading to early termination of the patient from the study, we will report the percentage of occurrence.

All predictable adverse events and adverse events need to be listed in the informed consent form and explain to the family. Unpredictable adverse events/adverse events need to be communicated to the family promptly after they occur and consent to continue the study. The occurrence of adverse events/adverse reactions needs to be judged by the attending whether to continue the current study and documented by the investigators to discuss whether the cause of occurrence is due to the study protocol for subsequent improvement.

Partially predictable adverse events are listed in the secondary outcomes.

## Trials status

Version number: 3, 2023 March 01.

This trial started recruiting patient since 2023 May 01. Sixty-eight infants will be recruited over 1 year. The trial will terminate when the last recruited infant is discharged from the hospital or dies.

### Supplementary Information

**Additional file 1.**

## Data Availability

Data sharing is not applicable to this article as no datasets were generated or analyzed during the current study. For the final study, datasets used and/or analyzed during this study are available upon reasonable request from the coordinating investigator. Data provided will consist of de‐identified participant with data dictionary, restricted to the data presented in the paper.

## Abbreviations

ABG: Arterial blood gas
BPD: Bronchopulmonary dysplasia
CIs: Confidence intervals
DSMB: Data safety monitoring board
ELBW: Extremely low birthweight
FiO_2_: Fraction of inhaled oxygen concentration
HFOV: High-frequency oscillatory ventilation
IVH: Intraventricular hemorrhage
MAP: Mean airway pressure
MV: Mechanical ventilation
NEC: Necrotizing enterocolitis
PaO_2_: Partial pressure of oxygen
PCO_2_: Partial pressure of carbon dioxide
PCV: Pressure control ventilation
PEEP: Positive end-expiratory pressure
PIP: Peak inspiratory pressure
PLV: Pressure limited ventilation
PVL: Periventricular leukomalacia
RCT: Randomized controlled trials
RDS: Respiratory distress syndrome
ROP: Retinopathy of prematurity
RR: Respiratory rate
SAE: Serious adverse events
SIMV: Synchronized intermittent mandatory ventilation
SpO_2_: Oxygen saturation
Ti: Inspiratory time
VG: Volume-guaranteed
VLBW: Very low birthweight
Vt: Tidal volume
VTV: Volume-targeted ventilation mode
